# Integrating dilution-based sequencing and population genotypes for single individual haplotyping

**DOI:** 10.1186/1471-2164-15-733

**Published:** 2014-08-28

**Authors:** Hirotaka Matsumoto, Hisanori Kiryu

**Affiliations:** Department of Computational Biology, Faculty of Frontier Science, The University of Tokyo, 5-1-5, Kashiwanoha, Kashiwa, Chiba, 277-8561 Japan

**Keywords:** Haplotype assembly, Single individual haplotyping, Statistical phasing, Dilution-based sequencing, Chimeric fragment

## Abstract

**Background:**

Haplotype information is useful for many genetic analyses and haplotypes are usually inferred using computational approaches. Among such approaches, the importance of single individual haplotyping (SIH), which infers individual haplotypes from sequence fragments, has been increasing with the advent of novel sequencing techniques, such as dilution-based sequencing. These techniques could produce virtual long read fragments by separating DNA fragments into multiple low-concentration aliquots, sequencing and mapping each aliquot, and merging clustered short reads. Although these experimental techniques are sophisticated, they have the problem of producing chimeric fragments whose left and right parts match different chromosomes. In our previous research, we found that chimeric fragments significantly decrease the accuracy of SIH. Although chimeric fragments can be removed by using haplotypes which are determined from pedigree genotypes, pedigree genotypes are generally not available. The length of reads cluster and heterozygous calls were also used to detect chimeric fragments. Although some chimeric fragments will be removed with these features, considerable number of chimeric fragments will be undetected because of the dispersion of the length and the absence of SNPs in the overlapped regions. For these reasons, a general method to detect and remove chimeric fragments is needed.

**Results:**

In this paper, we propose a general method to detect chimeric fragments. The basis of our method is that a chimeric fragment would correspond to an artificial recombinant haplotype and would differ from biological haplotypes. To detect differences from biological haplotypes, we integrated statistical phasing, which is a haplotype inference approach from population genotypes, into our method. We applied our method to two datasets and detected chimeric fragments with high AUC. AUC values of our method are higher than those of just using cluster length and heterozygous calls. We then used multiple SIH algorithm to compare the accuracy of SIH before and after removing the chimeric fragment candidates. The accuracy of assembled haplotypes increased significantly after removing chimeric fragment candidates.

**Conclusions:**

Our method is useful for detecting chimeric fragments and improving SIH accuracy. The Ruby script is available at https://sites.google.com/site/hmatsu1226/software/csp.

**Electronic supplementary material:**

The online version of this article (doi:10.1186/1471-2164-15-733) contains supplementary material, which is available to authorized users.

## Background

Advances in experimental techniques for DNA sequencing and genotyping have made it possible to determine many individual human genomes and detect variations, such as single nucleotide polymorphisms (SNPs) [1, 2]. This has brought about great progress in genome analyses, such as genome-wide association studies (GWAS) [3], inference of population structure [4], and expression phenotypes [5]. However, most technologies give only genotype information and most current research does not determine the haplotype origin of the variations. Haplotypes contain more detailed information than genotypes and are valuable for GWAS [6], and for analyzing genetic structures such as linkage disequilibrium, recombination patterns [1], and correlations between variations and diseases [7].

Determining haplotypes experimentally is difficult, and there are three main computational approaches for haplotype inference. The first approach is the statistical phasing method, which infers population haplotypes from population genotypes using statistical computation [8–12]. Algorithms for statistical phasing have been developed in response to technological advances for genotyping, and its basis is that the diversity of haplotypes is limited, and there are conserved haplotypes [13]. Because of the strategy, statistical phasing does not work well in chromosomal regions which exhibit several different haplotypes, particularly regions of low linkage disequilibrium. This approach is also weak in inferring long haplotypes because the complexity of population haplotypes increases exponentially according to the number of SNPs.

In the second approach, haplotypes are inferred from genotypes of pedigrees. For example, a child’s haplotypes are determined from the genotypes of child and its parents (trio-based haplotyping). The origin of child’s alleles can be determined if only one of the parents has the same alleles. However, the haplotypes of sites at which all family members have the same genotype cannot be determined and, furthermore, family genotype data are not always available. In addition, neither the statistical phasing method nor this approach can identify spontaneous mutations.

The third approach uses DNA sequencing data and is called single individual haplotyping (SIH) or haplotype assembly [14–22]. SIH utilizes the fact that each sequenced read is derived from only one of the haplotypes. If a read spans two or more heterozygous sites, the haplotype can be determined from the co-occurrence of alleles in the read. Two reads are determined to originate from the same chromosome if they overlap at a region that has at least one heterozygous site, and they have the same alleles at these sites.

**Figure 1:**
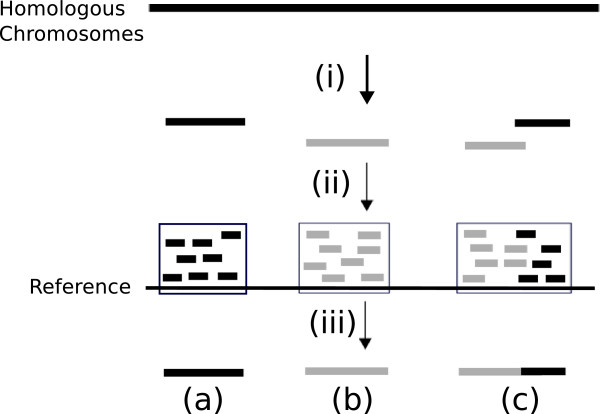
**An illustration of dilution-based sequencing.** (i) The DNA fragments are separated into multiple low-concentration dilutions. (ii) After sequencing and mapping an aliquot, mapped reads form clusters which correspond to DNA fragments. (iii) Clusters are merged into read fragments and result in natural fragments (a), (b) and a chimeric fragment (c). Chimeric fragments are produced when short reads derived from multiple DNA fragments are regarded as one cluster.

SIH did not attract much attention until recently, since it needed long DNA sequencing reads that spanned multiple heterozygous sites, and obtaining such reads quickly and economically was difficult. However, this situation is changing rapidly with the advent of new experimental techniques, such as fosmid pool-based next-generation sequencing [17, 23, 24], long read fragment technology [25], and dilution-amplification-based sequencing [26] that can produce virtual long reads. In these methods, long DNA fragments are separated into distinct low-concentration aliquots which each contain less than one fragment per chromosomal region. After sequencing an aliquot with a next-generation sequencer and mapping short reads, clusters are formed in which the reads are close to each other. A cluster corresponds to a long DNA fragment and is supposed to be derived from a single haplotype. Thus, virtual long reads can be obtained by merging the short reads in a cluster (see Figure 1).

Although such experimental techniques are sophisticated, they have the problem of producing chimeric fragments whose left and right parts match different chromosomes very well. Because long DNA fragments are separated into aliquots randomly, there are cases where an aliquot has some long DNA fragments derived from the same region of different chromosomes and, consequently, reads with different chromosomal origins are regarded as one cluster and merged into a single fragment (see Figure 1). In the process of developing MixSIH [22], which is the first SIH algorithm that can evaluate the reliability of a haplotype region, we have shown that such chimeric fragments significantly decrease the accuracy of SIH. This is because the chimeric fragments can lead to opposite haplotypes between right and left of haplotype regions.

In our previous study we detected chimeric fragments under the condition that parents genotypes were given. However, independence from pedigree data is one of the advantages of SIH and, therefore, it is common to assume that pedigree genotypes are not available. Even if pedigree genotypes are available, there are regions whose haplotypes are not determined from pedigree genotypes and the chimeric fragments in such regions cannot be detected with the previous method. The length of a reads cluster and heterozygous calls in a reads cluster were also used for detecting chimeric fragments [17]. The length of a reads cluster which correspond to a chimeric fragment will be larger than that of most reads clusters because reads with different long DNA fragment origins are regarded as one cluster and merged into one fragment. In addition, if there are some heterozygous SNPs in an overlapped region where reads with different haplotype origins coexist, these SNPs will show heterozygous calls in a reads cluster. Although some chimeric fragments will be detected with cluster length and heterozygous calls, considerable number of chimeric fragments will be left behind because of the dispersion of the cluster lengths, and non-detection of the heterozygous calls in the overlapped regions due to the lack of coverage and absence of heterozygous SNPs. For these reasons, chimeric fragment detection method which does not depend on pedigree genotypes and can detect chimeric fragments which are overlooked by the cluster length and the heterozygous calls is necessary to obtain high quality assembled haplotypes.

In this paper, we propose a general method to detect chimeric fragments without using pedigree genotypes. Our method is based on the assumption that chimeric fragments are derived artificially and differ from the biological conserved haplotypes in the population. Under this assumption, we use population genotypes to evaluate inconsistency between virtual long read and inferred haplotypes.

Previous researches showed that the quality of haplotype inference will increase by integration of SIH and statistical phasing [27–29]. These approach basically consider the SNP fragments as reliable information sources and use population haplotypes to supplement inferred individual haplotypes. Therefore, these approaches will not be suitable for preventing the effect of chimeric fragments, which are unreliable and can lead to incorrect haplotypes. Our research presents the importance of considering chimeric fragments in SIH and proposes a novel strategy for integration by focusing on the process of dilution-based sequencing.

We applied our method to two real datasets and showed that the chimeric fragments could be detected with high accuracy. Moreover, we compared the accuracy of multiple SIH algorithm for before and after removing chimeric fragments candidates. We found that accuracy of assembled haplotypes improved considerably after chimeric fragment candidates were removed using our method. In addition, we found that SIH algorithm successfully inferred long haplotypes and showed the usefulness of SIH.

## Methods

### Notation

Throughout the paper, we denote chimeric fragment as CF, and natural fragment as NF.

Because SIH is trivial for homozygous sites and because it is usually much easier to determine the genotype than to determine the haplotypes, we focus on heterozygous sites and represent heterozygous alleles by a simple binary representation. Fragments from which the homozygous sites have been removed are called SNP fragments. SNP fragments are represented by *F* = {*f*_*i*_|*i* = 1,…,*N*}, and fragment *f*_*i*_ takes value *f*_*ij*_ ∈ {0,1} at site *j* if *f*_*i*_ covers the site. We denote the set of sites which *f*_*i*_ covers by *X*(*f*_*i*_).

### Statistical phasing method

In this paper, we describe a method to detect CFs by using statistical phasing. The statistical phasing method estimates haplotypes from population genotype data based on the fact that the diversity of local haplotypes is low.

Here, we use the software PHASE (version 2.1.1) with default settings for statistical phasing [10, 11]. PHASE infers haplotypes of the specified set of SNPs *S* using the Gibbs sampling method which samples each individual in a random order, updates the individual haplotypes under the assumption that all the other haplotypes are given, and repeats this process. PHASE outputs several candidate haplotypes and their probabilities for each individual. In detecting CFs, we are interested in the individual haplotypes of the individual who is the target of SIH and denote the set of candidate haplotypes for the individual by , where *M* is the number of candidates and  is composed of the haplotype pair  and .  is composed of the set of  which represent the binary allele of the haplotype  at site *k*. We denote the probability of  for the individual by .

### Chimeric fragment detection model

We model probabilities that a fragment *f*_*i*_ is NF (*P*^*n*^(*f*_*i*_)) and CF (*P*^*c*^(*f*_*i*_)), and develop an indicator for detecting CF with these probabilities. Upon the calculation of the NF and CF probabilities of a fragment, we obtain *H*^(*p*)^ and  by running PHASE for *S* = *X*(*f*_*i*_).

The NF probability of fragment *f*_*i*_ is composed of the probability of the individual haplotypes and the probability of the SNP fragment given the haplotypes:


where *α* is a error term to deal with sequencing and PHASE errors. In this paper, we use *α* = 0.01 (the effect of changing *α* is described in the Additional file 1).

The CF probability is similar to the NF probability, but the probability of SNP fragments given haplotypes is slightly different.  is calculated by assuming that left and right parts of *f*_*i*_ are derived from different haplotypes in a haplotype pair:


where  and . Although we assume that the CF changes the origin of haplotype only once, it is possible that a CF changes the derivation many times over. However, such a CF would be rare and the CF probability given above would, in such a situation, approximate the result obtained by marginalizing over the switched sites.

Using these probabilities, we would like to define an indicator that evaluates the degree of artificiality of a recombinant SNP fragment which we will call the ‘chimerity based on statistical phasing’ (CSP). In principle, we would like to use


However, because the number of possible haplotypes and their combinations increase exponentially and the running time of PHASE increases according to SNP fragment size, we use a sliding-window approach to calculate CSP if the size of a SNP fragment is over sliding window width:


where  is the partial fragment of *f*_*i*_ which starts from the *β*th site and whose size is *W*. *X*^′^(*f*_*i*_) is *X*(*f*_*i*_) in which  is removed, where  is the rightmost partial fragment. *W* is the sliding window width and we use *W* = 5 for the default setting (see the Additional file 1 for the effect of changing *W*). In the process of sliding window calculation, *H*^(*p*)^ and  are obtained by running PHASE for .

We detect the CF candidates in a set of SNP fragments by selecting the SNP fragments whose CSP are larger than a threshold.

### Cluster length and heterozygous calls for detecting chimeric fragment

In the previous research, the length of a reads cluster and heterozygous calls in a reads cluster were used for filtering CFs [17]. Because a CF is produced when two long DNA fragments are regarded as one reads cluster, the length of reads cluster (cluster length) which corresponds to s CF tends to be larger than that of reads clusters which corresponds to NFs. Therefore, CFs can be detected by selecting the SNP fragments whose cluster length are over than a threshold. Moreover, if there are some heterozygous SNPs in a overlapped region and there are enough coverage, reads in a reads cluster will show heterozygosity. Because there are several evaluation for heterozygous calls in a reads cluster, we used three measure, the total number of reads which cover minority allele (total heterozygosity), maximum of the rate of the minority allele (maximum heterozygosity), and average of the rate of the minority allele (average heterozygosity) (see the Additional file 1 for the detailed definition). We compare the performance of CSP with that of methods based on cluster length and heterozygosity.

### Recovering SNP fragments from CF candidates

The CSP method might regard NFs as CF candidates when the NFs differ from population haplotypes due to rare variants or spontaneous recombination. To recover such NFs from CF candidates, we use coverage data. Because CFs are produced when an aliquot happens to contain some DNA fragments which cover the same regions, CFs would be distributed randomly. Therefore, if there are many CF candidates that cover the same regions, they would be NFs. We, therefore, recover the CF candidates which fulfill a coverage threshold condition. However, CFs might be accidentally located in a high coverage region and, therefore, we run SIH for recovered SNP fragments, calculate the chimerity based on inferred haplotypes, and remove SNP fragments whose chimerity is larger than a threshold. The detailed process and results are shown in the Additional file 1.

### Mixture model for SIH

We have previously developed a mixture model for SIH (MixSIH) [22]. Our model provides a confidence score for haplotype regions, and we could extract reliable haplotype blocks using this confidence score.

Here, we give a brief explanation of MixSIH. The probability distribution of the observed SNP fragments *F* were modeled by parameter Θ, which represents the phase of each site. *P*(*F*|Θ) can be represented by the indicator function that represents the haplotype origin of fragments. We used the VBEM algorithm to optimize Θ with the indicator function, and inferred haplotypes from optimized Θ.

In SIH, the associations in each segment are almost random if the number of connecting fragments is not sufficient or there are many conflicting fragments. Such sites often cause switch errors and, therefore, we need a method to evaluate the reliability of the connection of the haplotypes. With the optimized parameters, we defined the connectivity at site *j*_0_ as a ratio of the marginal log likelihoods:


where Θ^′^ correspond to a recombinant of Θ at site *j*_0_. The connectivity measures the resilience of the assembly result against swapping the two haplotypes at site *j*_0_.

We extended the idea of connectivity to give a confidence score for a region. For the region [*j*_1_,*j*_2_](*j*_1_ < *j*_2_), we defined the minimum connectivity (MC) sore as


We can extract reliable assembled blocks by selecting regions with high MC values.

### CF detection based on trio-based haplotypes

We defined the chimerity used to detect CF by using trio-based haplotypes in our previous research and use this indicator to define the true dataset.


where  is the pair of true haplotypes which are determined by trio-based haplotyping, *f*_*i*,≤*k*_ and *f*_*i*,>*k*_ represent the left and right parts of fragment *f*_*i*_ divided at site *k*, and *α*_0_ is the sequence error rate term. We define a CF as being an SNP fragment whose chimerity is over a threshold.

### Dataset and data processing

For the sequencing data, we used the data from Kaper et al. [26] and Duitama et al. [17]. Kaper and coworkers diluted and distributed long DNA fragments into physically distinct aliquots, while Duitama and coworkers partitioned long DNA fragments into distinct low-concentration aliquots using fosmid clones. The aliquots were sequenced using next-generation sequencers. After mapping the short reads onto the reference genome, short reads formed clusters in which the reads were close to each other. Each cluster corresponded to a long DNA fragment and was supposed to originate from the same haplotypes and, therefore, the alleles observed in a cluster could be merged into a SNP fragment. In the above procedure, CFs would be produced because an aliquot might contain some long DNA fragments derived from the same region of a different chromosome, and reads with different chromosomal origins might be merged into a single SNP fragment (Figure 1).

Both groups conducted analyses of the HapMap trio child NA12878 from the CEU population [1]. NA12878 had about 1.65 × 10^6^ heterozygous sites on an autosomal chromosome and the haplotypes of about 1.36 × 10^6^ sites were determined by a trio-based phasing method [2].

We aligned Kaper’s data and Duitama’s data to a human reference genome (hg18) using bowtie (version 1.0.0) and bfast (version 0.7.0), respectively. We identified read clusters that corresponded to long DNA fragments by using the targetcut function of SAMtools (version 0.1.19), and converted the clusters into SNP fragments by majority decision at the alleles of the heterozygous sites determined by the 1000 genomes project [2]. SNP fragments whose sizes were below 1 were discarded. Accordingly, 323,734 and 212,351 of SNP fragments were found for Kaper’s data and Duitama’s data, respectively. The average SNP fragment size in Kaper’s (Duitama’s) data was about 8.8 (22.6), and the average coverage of SNP fragments was 1.7 (2.9).

Next, we implemented filtering step for the reads cluster data to filter CFs by using the cluster length and heterozygous calls. This step is based on the preprocessing method proposed by previous research [17]. The reads cluster were divided into multiple reads clusters at the SNPs which show heterozygous calls. The heterozygous call was defined so that either one of the following two conditions were satisfied: (1) the number of reads which contain minority allele is larger than half the average coverage of the aliquot; (2) the number of reads which contain minority allele is larger than half of the number of reads which contain majority allele. The reads cluster which is significantly large (> 30 kb for Kaper’s data and > 45 kb for Duitama’s data) are divided into multiple reads cluster so that each cluster length is below threshold (30 kb and 45 kb, respectively). Accordingly, 346,417 and 436,543 of SNP fragments were found for Kaper’s data and Duitama’s data, respectively. The average SNP fragment size in Kaper’s (Duitama’s) data was about 8.0 (10.2), and the average coverage of SNP fragments was 1.7 (2.7). Hereafter, we designate this procedure as *filtering*.

In addition, we also used the original SNP fragments data of Duitama’s data which was downloaded from http://owww.molgen.mpg.de/~genetic-variation/SIH/data/. We designate this dataset as Duitama’s SNP fragments.

For statistical phasing, we used CEU population genotypes downloaded from the 1000 genomes project. To exclude the bias of related genotypes, the parents genotypes were removed. In total, 61 genotypes including NA12878 itself were used for PHASE. The influence of the number of individuals is discussed in the Additional file 1.

For SIH, we used ReFHap [17], FastHare [21], and DGS [19], which were implemented by Duitama [17] in addition to MixSIH.

### Accuracy measure for CF detection

To evaluate the detection of CFs by CSP, we defined true NFs and CFs by using chimerity. A true CF was defined to be an SNP fragment which satisfies chimerity ≥ 2 ln(*α*_0_/(1 - *α*_0_)), and a true NF was an SNP fragment which satisfies chimerity < 2 ln(*α*_0_/(1 - *α*_0_)). However, the chimerity of fragments for which haplotypes of the region could not be determined by trio-based haplotyping could not be calculated. For this reason, we removed such fragments from the evaluation. We defined sensitivity and specificity as the proportion of CFs which are detected and the proportion of the NFs which are detected by mistake, respectively.

Based on the chimerity threshold, the number of NFs and CFs in Kaper’s data (before filtering) are 283,270 and 6,864, respectively, while the number of NFs and CFs in Duitama’s data (before filtering) are 188,928 and 13,063, respectively. After filtering with cluster length and heterozygous calls, the number of NFs and CFs in Kaper’s data are 304,423 and 3,830, respectively, while the number of NFs and CFs in Duitama’s data are 384,857 and 6,381 respectively. The results of Duitama’s SNP fragments are shown in the Additional file 1.

The CF rate of Duitama’s data (before filtering) (6.5%) is larger than that of Kaper’s data (before filtering) (2.4%) because Duitama’s experimental approach tends to contain long DNA fragments from the same regions in a single aliquot, which results in CFs. Kaper separated long DNA fragments into 196 aliquots so that each aliquot would have a low concentration while Duitama separated fragments into 32 aliquots. Moreover, the DNA fragments in Duitama’s data are longer than those of Kaper’s data and the longer the DNA fragments are, the higher the probability that the DNA fragments overlap.

Although it is better for SIH to have fewer CFs, one cannot say unconditionally that Kaper’s data is better than Duitama’s data. This is because longer DNA fragments result in longer SNP fragments which are useful for assembling haplotypes. Moreover, from the perspective of efficiency and cost, separating long DNA fragments in more aliquots is difficult. For these reasons, each of the experimental approaches has merits and demerits.

### Accuracy measure for SIH

To evaluate the accuracy of the partially assembled haplotype, we defined a pairwise accuracy measure in previous research [22]. Let *H*^(*t*)^ be the true haplotypes, and  be the inferred haplotypes blocks. A pair of heterozygous sites *j* and *j*^′^ (*j* < *j*^′^) was defined as consistent if , and inconsistent otherwise, where  represents the allele of the *j*th locus belonging to the *i*th haplotype segment. For each haplotype block, we count the consistent and inconsistent pairs. The total numbers of consistent and inconsistent pairs over all the haplotype blocks are denoted by CP and IP, respectively. We defined *precision* by CP/(CP + IP). The detailed explanation is shown in previous research [22].

We also used other two accuracy measures, switch error rate and QAN50 [17]. The switch error rate is defined as the frequency of switch errors which are inconsistency between inferred haplotypes and true haplotypes. The QAN50 is remodeled from N50 so that it takes consistency between inferred haplotypes and true haplotypes into account. In short, prediction is divided into smaller haplotype blocks that do not contain any switch errors, and QAN50 is N50 of divided inferred haplotypes with some adjustments.

## Results and discussion

### Detection of chimeric fragments

**Figure 2:**
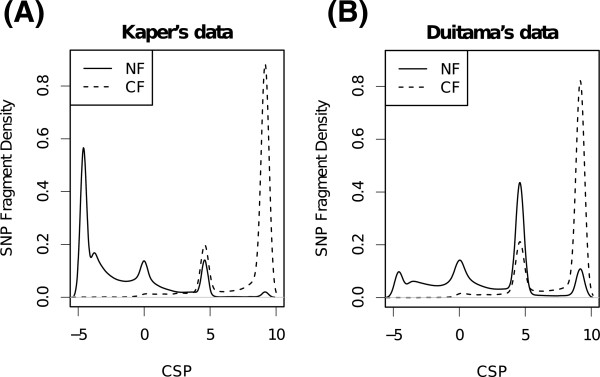
**Comparison of CSP density distributions for NFs and CFs. (A)** and **(B)** are the distributions of Kaper’s data and Duitama’s data, respectively.

We compared the CSP density distributions for NFs and CFs of the data before filtering (Figure 2). The CSP of CFs shows a tendency to be larger than that of NFs. This result suggests that the CFs are regarded as artificial recombinant haplotypes and hence differ from the biological haplotypes which exist in the population. There are peaks in the CSP density distributions at 4.6 and 9.2. These peaks correspond to SNP fragments which are inconsistent with statistically phased haplotypes and are consistent when the SNP fragment changes the derivation to another haplotype. The CSP is around 4.6 (≈- ln(*α*/(1 - *α*)) when a SNP fragment changes the haplotype origin at the first site from the end, and the CSP is around 9.2 (≈ - 2 ln(*α*/(1 - *α*)) when a SNP fragment changes the haplotype origin at the second site from the end. For W = 5, the CSP of CFs which are inconsistent with statistically phased haplotypes is expected to be around 9.2 because in that case the SNP fragment is recombinant at the second site from the end in the sliding window calculation. Actually, 74.1% (71.9%) of CFs in Kaper’s (Duitama’s) data are between CSP = 7 and CSP = 12, and 1.5% (9.7%) of NF are within the same bounds. The peak at 4.6 is likely to be caused by sequencing and statistical phasing errors.

**Figure 3:**
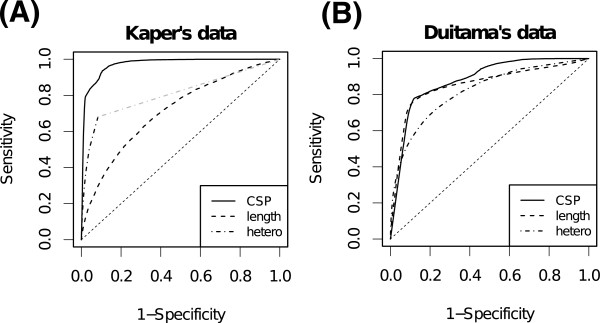
**The ROC curves of CSP, cluster length, and total heterozygosity for classification of CFs and NFs.** The ROC curves are obtained by changing the threshold of CSP, cluster length, total heterozygosity, respectively. There is a region that the data point of the ROC curve of total heterogeneity for Kaper’s data is absent, and hence, the ROC curve is supplemented (shown as gray line). **(A)** and **(B)** correspond to Kaper’s data and Duitama’s data, respectively.

**Figure 4:**
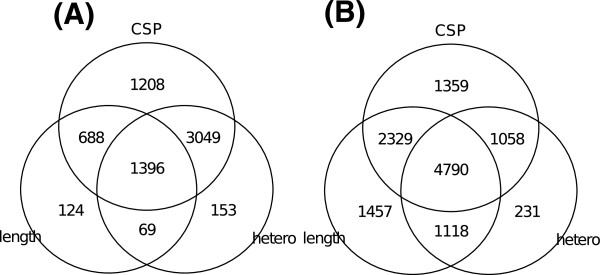
**The Venn diagrams of CFs detected by CSP, length, and total heterozygosity.** The number in each cell is the number of CFs in the corresponding category. The threshold for CF detection of each measure was set so that the 1-specificity was under 0.1. **(A)** and **(B)** correspond to Kaper’s data and Duitama’s data, respectively.

Figure 3 shows the ROC curves of CSP, cluster length, and total heterozygosity for each dataset before filtering. The ROC curves of maximum heterozygosity and average heterozygosity are inferior to that of total heterozygosity, and are shown in the Additional file 1. The area under the curve (AUC) of CSP for Kaper’s data is 0.97 and the AUC for Duitama’s data is 0.88. These values are higher than those of cluster length (0.71 for Kaper’s data and 0.85 for Duitama’s data) and total heterozygosity (0.80 for Kaper’s data and 0.82 for Duitama’s data). The AUC values of cluster length are lower than that of CSP, especially in the case of Kaper’s data, and this is because the cluster length of NFs and CFs overlap significantly (see the Additional file 1 for the distribution of cluster length of NFs and CFs). The AUC values of total heterozygosity are lower than that of CSP and this is because there are considerable CFs which do not show heterozygosity due to the lack of coverage and absence of heterozygous SNPs in overlapped regions. Moreover, sequencing error will disturb to distinguish NFs and CFs because sequencing errors in NFs will bring heterozygous calls and such NFs might be regarded as CFs by mistake. These results show the high performance of the detection of CFs using CSP, regardless of the experimental conditions. The difference between the AUC values of CSP for each dataset might be caused by the error rate in SNP fragments; The SNP fragment error rate of Duitama’ data is 4.0% and that of Kaper’s data is 1.2% (see the Additional file 1 for the SNP fragment error rate calculation).Figure 4 shows the Venn diagrams of CFs detected by CSP, cluster length, and total heterozygosity for each dataset. The threshold of each measure was set so that (1-specificity) was under 0.1. In Kaper’s data, the number of CFs which were detected with CSP was largest, and about 94% of CFs which were detected with either cluster length or total heterozygosity were also detected with CSP. In Duitama’ data the number of CFs which were detected with CSP was slightly lower than that of CFs detected with cluster length, but about 14% of CFs detected with CSP were detected with neither cluster length nor total heterozygosity. These results also show that CSP is an effective indicator for detecting CFs which are detected with neither cluster length nor heterozygosity. Since there are significant number of CFs which are detected only with cluster length and heterozygosity calls, we compare the SIH accuracies of the SNP fragments that are filtered with cluster length and heterozygous calls with those of the SNP fragments that are further filtered with CSP, and examined the usefulness of CSP in SIH in the following section.

### SIH accuracy after removing suspicious CFs by using CSP

We defined a CF candidate as a SNP fragment whose CSP was larger than 7, and removed these from SNP fragments. We hereafter represent the SNP fragments filtered with cluster length and heterozygous calls as "filtered", and the SNP fragments further filtered with CSP as "filtered+CSP". The CSP threshold was determined so that many CFs were removed while avoiding a high false-positive rate; many CFs had a CSP of around 9.2 and there were many NFs with around CSP = 4.6 (Figure 2). With this procedure, 1.6% (5,375/346,417) of Kaper’s data and 3.8% (16,715/436,543) of Duitama’s data were removed. The removed fragment rate for Duitama’s data was higher than that for Kaper’s data because Duitama’s data would contain more CFs because of the experimental approach (see Section ‘Dataset and data processing’ for a detailed explanation).

**Figure 5:**
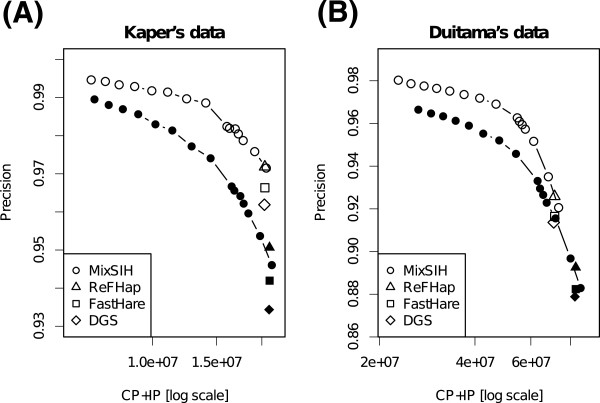
**Precision curves based on consistent pair counts.** The *x*-axis represents the number of predicted pairs on a log scale. MC of MixSIH was changed from 0 to 10. The accuracies of the data filtered with cluster length and heterozygous calls (filtered) (filled point symbols) and the further filtered data, in which fragments with CSP > 7 are removed (filtered+CSP) (empty point symbols), are shown for Kaper’s data **(A)** and Duitama’s data **(B)**: ∘ MixSIH; △ ReFHap; □ FastHare; ◇ DGS.

Figure 5 shows the accuracies of MixSIH, ReFHap, FastHare, and DGS for each dataset: filtered with cluster length and heterozygous calls (filtered); further filtering with CSP (filtered+CSP). The precision of MixSIH increased from about 0.972 to 0.985 at (CP + IP) = 1.5 × 10^7^ for Kaper’s data, and increased from about 0.950 to 0.966 at (CP + IP) = 5.0 × 10^7^ for Duitama’s data. The precision of other algorithm increased likewise. In addition, the precision for Duitama’s SNP fragments also increased after removing CFs candidates with CSP (shown in the Additional file 1). Thus, CSP increases SIH accuracy by removing CF candidates which would have a serious influence.

In addition, (CP+IP) for Duitama’s data is larger than that for Kaper’s data because the SNP fragment size and coverage are larger. The precision of Kaper’s data is higher because it contains fewer CFs and the SNP error rate is lower; the decrease of (CP+IP) is lower for the same reason.

Tables 1 and 2 show the switch error rate and the QAN50 of each algorithm for each dataset, respectively. In these analyses, MC of MixSIH were set to 10. The switch error rate improved after removing suspicious CFs in all conditions. This result is consistent with the result based on pairwise accuracy measure and shows the usefulness of removing CFs with CSP. Switch error rates of MixSIH were lowest in all conditions and this suggests that MixSIH succeeds to extract reliable haplotype regions with MC values.

**Table 1:** **The switch error rate (%) of each SIH algorithm for data (filtered) and data (filtered+CSP)**

		MixSIH	ReFHap	FastHare	DGS
(A)	filtered	0.67	1.54	1.59	1.73
	filtered+CSP	0.52	1.22	1.28	1.38
(B)	filtered	2.75	3.22	3.28	3.47
	filtered+CSP	2.13	2.77	2.84	3.03

MC of MixSIH is set to10. (A) and (B) correspond to Kaper’s data and Duitama’s data, respectively.

**Table 2:** **QAN50 (kb) of each SIH algorithm data (filtered) and data (filtered+CSP), in which fragment with CSP > 7 are removed**

		MixSIH	ReFHap	FastHare	DGS
(A)	filtered	16.6	27.3	27.1	26.8
	filtered+CSP	16.6	27.5	27.4	27.2
(B)	filtered	32.7	69.2	68.4	67.7
	filtered+CSP	32.5	70.4	70.0	68.6

MC of MixSIH is set to10. (A) and (B) correspond to Kaper’s data and Duitama’s data, respectively.

The QAN50 also improved after removing suspicious CFs in all conditions excluding the QAN50 of MixSIH at MC = 10. The QAN50 of MixSIH at MC = 10 were lowest in those of other algorithm and did not improve after removing CF candidates. This is because QAN50 does not contain the penalty of connecting wrong haplotypes and will improve just by connecting two haplotypes blocks randomly with probability 0.5, and is inappropriate to evaluate extracting reliable haplotypes.

From these results, we concluded that CSP is an efficient indicator to improve SIH accuracy by removing suspicious CFs.

### Assembled haplotype block size

We examined the size distribution of assembled haplotype blocks. The haplotypes were inferred from each dataset in which the fragments with CSP larger than 7 were removed. Table 3 shows the number of haplotype blocks that contain the certain range of the number of phased SNPs for each dataset. For comparison, the number of SNP fragments that cover the certain range of the number of SNPs are also shown.

**Table 3:** **The number of the SNP fragments which cover the certain range of the numbered of SNPs (before SIH) and the number of haplotype blocks which contain the certain range of the number of phased SNPs (after SIH)**

		–10	11–20	21–50	51–100	101–200	201–
(A)	Before SIH	261,537	65,429	18,894	540	16	1
	After SIH	28,631	10,503	11,186	3,998	923	72
(B)	Before SIH	291,495	92,104	49,092	3,652	192	8
	After SIH	15,273	4,037	6,039	4,882	3,267	1,202

(A) and (B) correspond to Kaper’s data and Duitama’s data, respectively. Note that a SNP can be contained in multiple SNP fragments and the halotype blocks do not overlap each other. The first row defines the range of the number of SNPs.

The averages of haplotype block size are about 19.2 and 42.6 for Kaper’s data and Duitama’s data, and they are larger than the averages of SNP fragment size (8.0 and 10.2, respectively). Moreover, the number of haplotype blocks that contain more than 100 SNPs are larger than the number of SNP fragments for both dataset. These results suggest that MixSIH succeeds to assemble haplotypes from SNP fragments. 1.8% and 12.9% of haplotype blocks in Kaper’s data and Duitama’s data contain more than 100 phased SNPs, and the ratio of phased SNPs in such long haplotype blocks to total SNPs are about 13.1% and 53.8%, respectively. This result suggests that SIH is able to determine long haplotypes which are not determined by statistical phasing.

In addition, the haplotype blocks in Duitama’s data tend to be longer than those of Kaper’s data because the SNP fragment size and coverage are larger. This result shows that SIH will be able to infer longer haplotypes in accordance with improvements of sequencing technologies.

### Comparison of MixSIH and PHASE

The strong and weak points of SIH and statistical phasing will differ because they use different information for inferring haplotypes. For example, SIH cannot infer haplotype regions which lack SNP fragments because of sequencing and mapping difficulties. Statistical phasing is weak in determining haplotype regions where linkage disequilibrium values are high and there are multiple haplotypes in population. To investigate these differences, we compared the reliabilities of MixSIH and PHASE.

**Figure 6:**
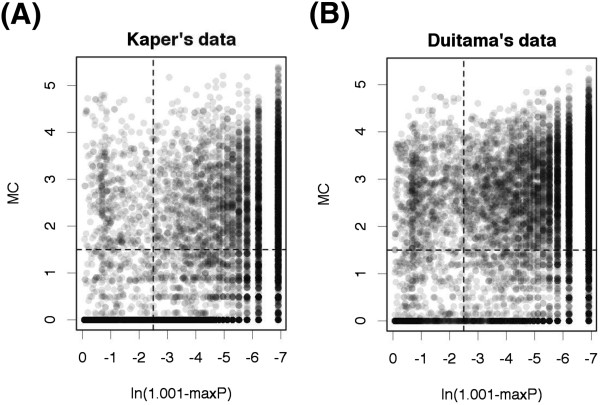
**Comparison of MC scores and maximum PHASE probabilities. (A)** and **(B)** correspond to Kaper’s data and Duitama’s data, respectively. The *x*-axis represents ln(1.001- max *P*), where max *P* is the maximum PHASE probability and we use 1.001 to deal with max *P* = 1.0. The *y*-axis represents the MC score of MixSIH. Data are randomly selected 1000 times from chromosome 1. The vertical dotted line corresponds to the maximum PHASE probability above which the precision of PHASE is over 0.9, and the horizontal dotted line corresponds to the MC value above which precision of MixSIH is over 0.9.

We selected 10,000 regions in chromosome 1 randomly so that each region had five SNP sites and the haplotypes of the regions were determined by trio-based haplotyping. We used Kaper’s data (filtered) and Duitama’s data (filtered) for SIH in this section. Figure 6 shows the MC value and the maximum probability of the PHASE for each region. The *x*-axis is ln(1.001- max*P*), where max*P* is the maximum haplotypes probability of PHASE for the region. We used 1.001 to deal with the case that max*P* = 1.0. The vertical dotted line corresponds to the maximum probability above which the precision of PHASE is over 0.9, and the horizontal dotted line corresponds to the MC value above which precision of MixSIH is over 0.9 (see the Additional file 1 for the calculation of precision).

Table 4 shows the number of regions for each division created by the previously noted dotted lines. In Duitama’s data, the rates in upper left division and lower right division are 8.4% and 22.2%, respectively. This result suggests that there are chromosomal regions for which SIH successfully infers the haplotypes and statistical phasing fails, and vice versa. The rate in the lower right division of Duitama’s data decreases from 22.2% to 14.1% when we remove the regions which contain sites that lack SNP fragments. This result suggests that many regions where SIH does not work are the result of a lack of SNP fragments.

**Table 4:** **The numbers of regions for each of the areas which are defined by the precision of MixSIH and PHASE: (A) Kaper’s data and (B) Duitama’s data**

		PHASE < 0.9	PHASE ≥ 0.9
(A)	MixSIH ≥ 0.9	433 (366)	3,499 (2,792)
	MixSIH < 0.9	1,096 (251)	4,972 (988)
(B)	MixSIH ≥ 0.9	842 (749)	6,250 (5,337)
	MixSIH < 0.9	687 (390)	2,221 (1,061)

The rows and columns represent the accuracy of MixSIH and PHASE, respectively. The numbers in parentheses are the numbers of regions remaining after regions which contain sites that lack SNP fragments have been removed.

Moreover, the rate in the upper divisions for Kaper’s data and Duitama’s data are 39.3% and 70.9%, respectively. The rate for Duitama’s data is larger than that for Kaper’s data because SNP fragment size and coverage are larger. This result suggests that SIH results will be improved just by getting larger and more SNP fragments.

In summary, there are regions where either SIH or statistical phasing can infer the haplotypes for these data. In the case of SIH, a shortage of data is likely to be the main reason for inference failure. For this reason, the performance of SIH will increase with advances in sequencing techniques.

## Conclusions

In this paper, we have developed a general method to detect chimeric fragments (CFs) on the assumption that CFs correspond to an artificially recombinant haplotype and differ from the biological haplotypes in the population. Based on this assumption, we developed natural fragment (NF) and CF probabilities of a fragment which use the result of statistical phasing. The NF probability calculates the consistency between a fragment and statistically inferred haplotypes. The CF probability also calculates the consistency, but it assumes that left and right parts of the fragment are derived from different haplotypes in a haplotype pair. With these probabilities, we developed an indicator CSP which evaluates the degree of chimerity by calculating the logarithmic difference.

We applied CSP to two sequencing datasets, Kaper’s data and Duitama’s data [17, 26]. The CSP of CFs tends to be lower than that of NFs Moreover, there are a lot of CFs at around possible largest value. These results support the propriety of our model. The high AUC values of CSP (0.97 for Kaper’s data and 0.88 for Duitama’s data) also shows that CSP is a highly efficient measure to detect CFs. The AUC values of CSP are higher than that of measures based on cluster length and heterozygosity. Moreover, there are significant number of CFs which are only detected with CSP. These results suggests the usefulness of CSP for detecting CFs.

We then compared the accuracies of MixSIH before and after removing the chimeric fragment candidates detected using CSP. The accuracies of MixSIH increased significantly after removing CFs. From these results, we conclude that CSP is a useful method for detecting CFs and improving SIH accuracy, regardless of the type of dilution-based sequencing.

In addition, we analyzed the results of MixSIH. The assembled haplotype blocks contain a lot of long haplotype blocks and this supports the capability of SIH that SIH can determine long haplotypes. We also compared the performance of MixSIH and statistical phasing method (PHASE). At the moment, the number of correctly inferred regions of PHASE is larger than that of MixSIH. However, lack of SNP fragments is the main reason for failure of SIH and, therefore, the importance of SIH and our method will increase in accordance with the advance of sequencing technologies.

In the future the amount of dilution-based sequencing data will increase, and our approach will be an important strategy not only for SIH but also for many other types of analysis, such as the detection of novel recombinant events.

## Electronic supplementary material

Additional file 1: **Supplementary text.** This file includes the explanation of parameter selection and some additional analyses. (PDF 116 KB)

## Abbreviations

SIH: Single individual haplotyping
CF: Chimeric fragment
NF: Naturel fragment
CSP: Chimerity based on statistical phasing
CP: consistent pair
IP: inconsistent pair
MC: Minimum connectivity
AUC: Area under the curve.
