# Supplementary material for: Integrating dilution-based sequencing and population genotypes for single individual haplotyping
Source: BMC Genomics. 2014 Aug 28;15(1):733. doi: 10.1186/1471-2164-15-733 (PMC4162929; doi:10.1186/1471-2164-15-733)
Supplement: Supplementary file 1 — Additional file 1: Supplementary text. This file includes the explanation of parameter selection and some additional analyses. (PDF 116 KB) [file 12864_2013_6431_MOESM1_ESM.pdf]

Supplementary Text for Integrating dilution-based sequencing and population genotypes for single individual haplotyping

Hirotaka Matsumoto, Hisanori Kiryu

June 13, 2014

1 Cluster length and heterozygous calls

1.1 Evaluation of heterozygous calls in a reads cluster

To detect CF by using the heterozygous calls in a reads cluster, we defined three measurements to evaluate the heterozygosity of SNP fragment f_i.

Firstly, we defined the total number of reads which cover minority allele (total heterozygosity) as follows:

$$\sum_{j \in X(f_i)} \min(n(r_{i,j} = 0), n(r_{i,j} = 1)) \ ,$$

where $n(r_{i,j} = 0)$ and $n(r_{i,j} = 1)$ are the number of reads, which are contained in a reads cluster which corresponds to a SNP fragment f_i and whose base at j-th locus are major allele and minor allele, respectively.

Secondary, we defined maximum of the rate of the minority allele (maximum heterozogosity) as follows:

$$\max_{j \in X(f_i)} \frac{\min(n(r_{i,j} = 0), n(r_{i,j} = 1))}{n(r_{i,j} = 0) + n(r_{i,j} = 1)} \ .$$

Thirdly, we defined average of the rate of the minority allele (average heterozygosity) as follows:

$$\frac{1}{|X(f_i)|} \sum_{j \in X(f_i)} \frac{\min(n(r_{i,j} = 0), n(r_{i,j} = 1))}{n(r_{i,j} = 0) + n(r_{i,j} = 1)} \ .$$

With these measurements, we detected CFs candidates by selection the fragments whose values are larger than a threshold.

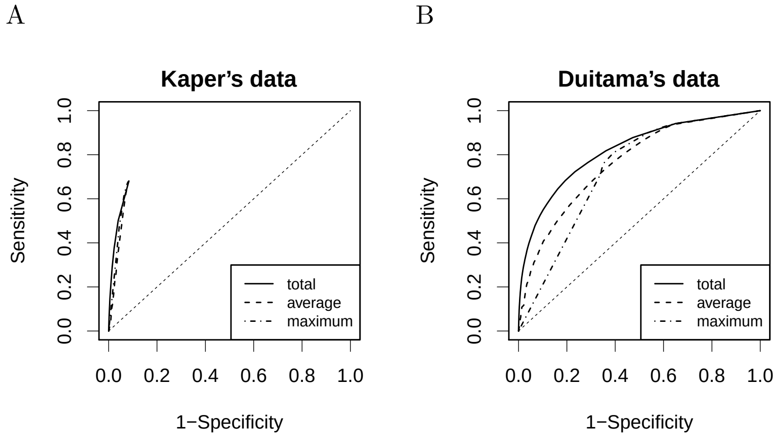

Figure 1: The ROC curves of total heterozygosity, average heterozygosity, and maximum heterozygosity for classification of CFs and NFs. A and B correspond to Kaper's data and Duitama's data, respectively.

1.2 ROC curves of heterozygosity evaluation

Figure 1 shows the ROC curves of total heterozygosity, maximum heterozygosity, and average heterozygosity. In Kaper's data, ROC curves stops at sensitivity is around 0.7. This is because there are many CFs which do not show heterozygous, and this could be caused when the coverage is low and only one origin of reads which derived from the same haplotype exist. In Duitama's data, the ROC curve of maximum heterozygosity and averaged heterozygosity are below that of total heterozygosity. This is because maximum and average heterozygosity overestimate the effect of sequencing error. Therefore, we concluded that total heterozygosity is appropriate to evaluate heterozygosity in a reads cluster.

1.3 Distribution of length of reads clusters

Figure 2 shows the distribution of the length of reads clusters for each dataset. The length of reads cluster which correspond to CFs tend to be larger because reads with different long DNA fragments origins are merged into one reads cluster. Although the cluster length of CFs tend to be larger than that of NFs, there are considerable overlapping between NFs and CFs, especially in the Kaper's data.

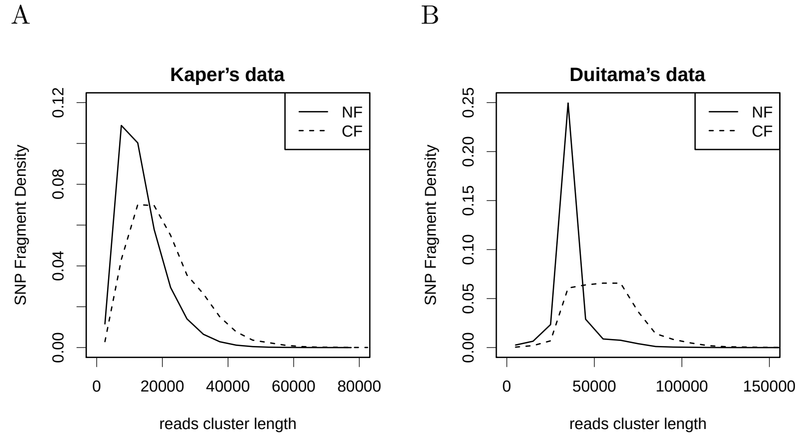

Figure 2: The distribution of cluster length. The x-axis represents the length of reads cluster and the y-axis represents the number of SNP fragments which are correspond to the each reads cluster.

2 Effects of changing various parameters

2.1 Impact of changing sliding window width on accuracy and running time

PHASE takes time to deal with a long SNP fragment because the number of possible haplotypes and their combinations increases exponentially. We defined a sliding window calculation to reduce the running time for long fragments. Because the sliding window width would affect the result, we examined the impact of sliding window width (W) on accuracy and running time. We used SNP fragments of chromosome 1 from Kaper's data for the AUC calculation, and used 100 randomly generated SNP fragments of size 30 for the running time calculation.

Figure 3 shows the AUC values and running times for W=3, 5, 7, 9. AUC increases roughly in line with the increase of W. This is because the difference between haplotypes becomes clearer when we consider more SNPs. However, difference between AUC values for W=3 and W=5 is larger than that for W=5 and W=7, which suggests that AUC would roughly saturate for low W. Running time also increases with increasing W. This is because the possible haplotypes and combinations of haplotypes increase exponentially as W increases. In view of these accuracy and running time results, we use W=5 as the default setting.

2.2 Effect of error rate α

We included an error term in CSP to represent sequencing and PHASE errors. To examine the effect of the error rate parameter α, we calculated AUC values

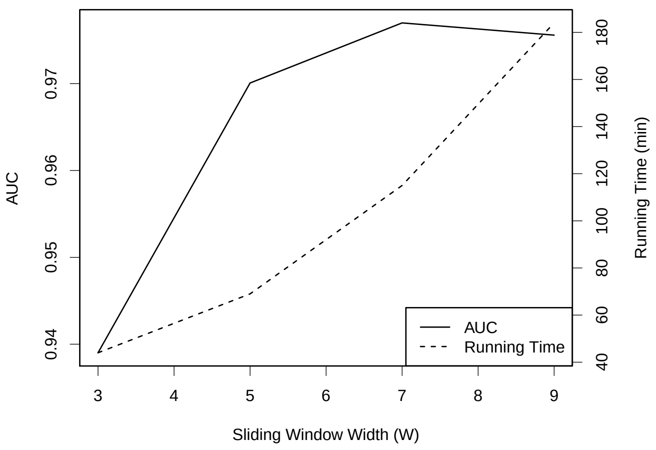

Figure 3: AUC values and running times for various values of W.

for various values of α. We used chromosome 1 from Kaper's data and Duitama's data for the AUC calculation. Table 1 shows the AUC values for each α. The AUC for $\alpha = 0.0$ is lowest because CSP with $\alpha = 0.0$ cannot deal with the inconsistency between inferred haplotypes and the context of a fragment which is caused by the sequencing and PHASE errors. The AUC values for $0.001 \leq \alpha \leq 0.1$ are almost equal. These results suggest that including α in CSP is important but the absolute value of α is unimportant. Based on these results, we use α=0.01 as the default value.

Table 1: AUC values for each α

	Kaper's data	Duitama's data
α=0.0	0.724	0.683
α=0.001	0.970	0.879
α=0.01	0.970	0.878
α=0.1	0.969	0.882

2.3 Effect of the number of individual genotypes

The accuracy of PHASE should increase with the number of individual genotypes. To examine the effect of changing the number of individual genotypes, we calculated the AUC of CF detection using chromosome 1 from Kaper's data and selecting N=5, 10, 20, 40, 60 individuals randomly from 60 unrelated individuals in the CEU population. We ran PHASE for randomly selected genotypes and the NA12878 genotype, and calculated AUC using the result of PHASE (Figure 4). AUC increases with the number of individuals. However, the rate

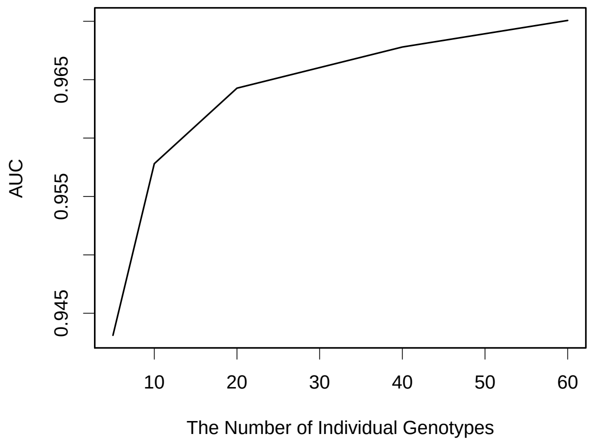

Figure 4: AUC values for various numbers of individuals.

of increase slows when the number of individuals increases. This suggests that detecting CFs which are located in multiple haplotype regions or contain sequencing errors, is difficult regardless of the number of individuals.

3 Recovering SNP fragments from CF candidates

CSP might regard NFs as CF candidates when NFs differ from population haplotypes because of rare variants or spontaneous recombination. As CFs are generated because an aliquot occasionally contains multiple DNA fragments which cover the same region, CFs would be distributed randomly. Therefore, if there are many CF candidates which cover the same region, they would be misidentified NFs. Because some CFs remain with only the threshold coverage, we removed fragments using a SIH-based measure. The detailed process is as follows:

1. Calculate the coverage of CF candidates for each heterozygous site.
2. Exclude sites whose coverage is lower than 3 and recover the SNP fragments which correspond to the remaining sites (P1).
3. Run MixSIH for recovered SNP fragments.
4. Calculate the chimerity-like measure 'SIH-chimerity'

$$\text{SIH-chimerity}(f) = -\ln\left(\frac{\max_{i=0,1} P^t(f|\hat{H}_i)}{\max_{i=0,1,j\in X(f)} P^t(f_{\le j}|\hat{H}_i)P^t(f_{>j}|\hat{H}_{\bar{i}})}\right) ,$$

Table 2: The numbers of all fragments, NFs, and CFs after performing each process on Kaper's data (A) and Duitama's data (B).

A

	Before	P1	P2
All	5,375	290	288
NF	1,924	236	235
CF	3,030	6	5

B

	Before	P1	P2
All	16,715	4,151	4,045
NF	10,699	2,759	2,692
CF	4,875	897	858

where $\hat{H} = (\hat{H}_0, \hat{H}_1)$ is the pair of haplotypes which are inferred by MixSIH.

5. Remove the fragments which satisfy SIH-chimerity $\geq 2\ln(\alpha_0/(1-\alpha_0))$ (P2).

Table 2 shows the numbers of all fragments, NF, and CF before and after recovery. The numbers of all fragments are larger than sums of NFs and CFs because trio-based haplotyping is partial and the chimerity of fragments which cover unphased regions cannot be calculated. The rates of CF for Kaper's data are 61.2%, 2.5%, and 2.1%, and the rates of NF for Duitama's data are 31.3%, 24.5%, and 24.2%. For both of datasets, the rates of CF decrease and we successfully recover NFs from CF candidates with high precision. The recovered fragments rates are 4.4% (235/5,375) and 16.1% (2,692/16,715) for Kaper's data and Duitama's data, respectively. The rate of recovered fragments for Duitama's data is larger than that for Kaper's data because the coverage of Duitama's data is higher than that of Kaper's data. High coverage might result in a larger CF rate in recovered fragments for Duitama's data.

In summary, NFs could be recovered from the CFs candidates by using the coverage information and SIH based chimerity. The coverage threshold should be determined according to the purpose of the analysis because there is a tradeoff between sensitivity and specificity.

4 Calculation of SNP fragment error rate

The SNP fragment error rate was calculated by comparison with the results of trio-based haplotyping. Because we were interested in the SNP fragment errors which were caused by sequencing and mapping errors, and CFs might disrupt the error rate calculation, we used only SNP fragments whose chimerity was under $2\ln(\alpha_0/(1-\alpha_0))$ for the calculation. The SNP fragment error rate is

$$\frac{\sum_{i=1}^{N} \min_{j=0,1}\left(\sum_{k\in X'(f_i)} I(f_{ik} \neq H_{jk}^{(t)})\right)}{\sum_{i=1}^{N} |X'(f_i)|},$$

where $X'(f_i)$ is the set of sites which are covered by f_i and whose phases are determined by trio-based haplotyping, $|X'(f_i)|$ is the number of sites in $X'(f_i)$,

Table 3: The number of NFs and CFs of Duitama's SNP fragments (A) and our processed Duitama's data (B).

	NF	CF
(A)	245,772	8,247
(B)	384,857	6,381

and $I(f_{ik} \neq H_{jk}^{(t)})$ is 1 when f_{ik} is inconsistent with reference haplotype $H_{jk}^{(t)}$ and 0 otherwise.

5 Comparison for Duitama's SNP fragments

5.1 The number of NFs and CFs of Duitama's SNP fragments

The number of NFs and CFs of Duitama's SNP fragments are 245,772 and 8,247, respectively, while the number of NFs and CFs of our processed Duitama's data are 384,857 and 6,381, respectively (Table 3). The number of NFs of Duitama's SNP fragments is lower than that of our data. This difference could be caused by the mapping tools, the reads cluster detection algorithm, and the filtering step. We used bfast for mapping SOLiD reads instead of BioScope which was used by Duitama et al. because the original bfast paper suggested that bfast has robustness against the sequence variants, and BioScope was not easily available. We used the targetcut function of the SAMtools which was used by Kaper et al. for reads cluster detection because the source code of cluster detection used by Duitama et al. was not open.

Concerning that the number of CFs of our data is lower than that of Duitama's SNP fragments, our processing method turns out to be more strict processing method. Some reads clusters will be divided into smaller reads clusters with the strict processing method, and this results in the increase of the number of NFs.

The SIH accuracy was shown to decrease with the presence of CFs. Therefore, our processing method which generates less CFs will be better than Duitama's processing method in terms of SIH accuracy.

5.2 SIH accuracy of Duitama's SNP fragments after removing suspicious CFs by using CSP

The SNP fragments data, in which long reads cluster and heterozygous calls are already filtered, is open by Duitama's group and we examined the pairwise accuracies of original Duitama's SNP fragments and processed Duitama's SNP fragments, in which fragments with CSP > 7 are removed (Figure 5 (A)). For comparison, the pairwise accuracies our processed Duitama's data that are already shown in the main text are shown again (Figure 5 (B)). With the CSP filtering procedure, 4.6% (12,364/271,184) of Duitama's SNP fragments were

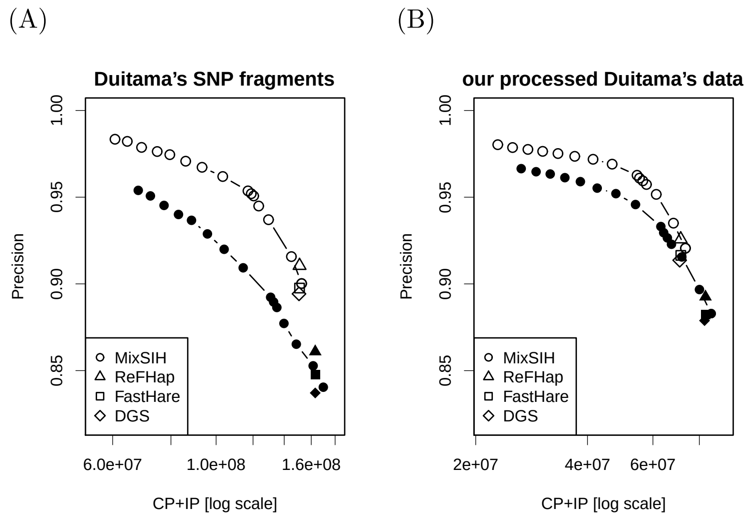

Figure 5: Precision curves based on consistent pair counts for Duitama's SNP fragments (A) and our processed Duitama's data (B). The x-axis represents the number of predicted pairs on a log scale. MC of MixSIH was changed from 0 to 10. The accuracies of the original data (filled point symbols) and the processed data (empty point symbols), in which fragments with CSP > 7 are removed, are shown: $\circ$ MixSIH; $\triangle$ ReFHap; $\Box$ FastHare; $\diamond$ DGS.

removed. The precision of MixSIH increased from 0.875 to 0.925 at (CP+IP) $= 1.4 \times 10^8$. The precision of other algorithm increased likewise. Thus, CSP is an efficient measure to detect the CFs which are undetected with cluster length and heterozygous calls, and useful for improving SIH accuracy.

In addition, (CP+IP) for Duitama's SNP fragments is larger than that for our processed Duitama's data, while the precision of each algorithm for Duitama's SNP fragments are lower than those for our data. These differences are caused by the difference of the processing methods (as discussed in the above section). With the strict processing method, the length of SNP fragments become smaller owing to the division of the reads cluster, and hence the length of assembled haplotypes is smaller. On the other hand, the strict processing method generates less CFs and the precision of assembled haplotypes increase.

6 Precision of MixSIH and PHASE

The precision of MixSIH was calculated as follows.

1. Select 10,000 regions in chromosome 1 randomly such that each region has five SNP sites and the haplotypes of the regions are determined by trio-based haplotyping.

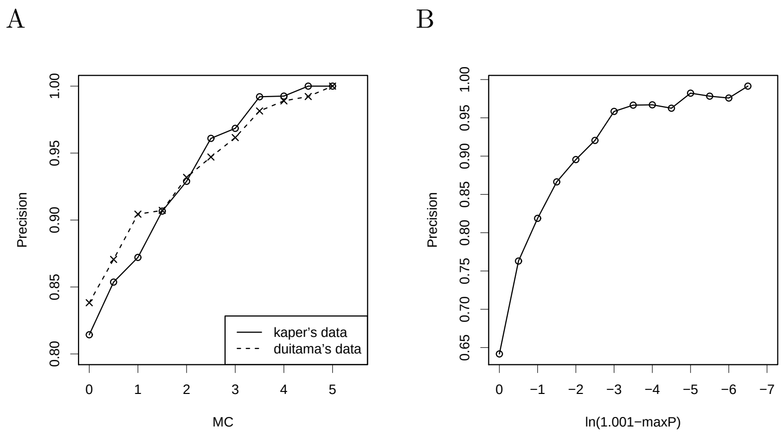

Figure 6: The consistent pair precision of (A) MC value and (B) maximum PHASE probability (maxP) for 5 SNPs regions.

2. Calculate MC values for each region.
3. Calculate the precision for MC value, which is defined by

$$\mathrm{CP}_{mc}/(\mathrm{CP}_{mc} + \mathrm{IP}_{mc}) ,$$

where mc is the target MC value, and CP_{mc} and IP_{mc} are the number of consistent pairs and inconsistent pairs in the regions for which MC value satisfy $mc \leq \mathrm{MC} < mc + 0.5$.

Figure 6(A) shows the precision for each dataset. In our evaluation, MixSIH precisions are over 0.90 for MC ≥ 1.5.

The precision for each $\ln(1.001 - \max P)$, where $\max P$ is the maximum PHASE probability, was calculated as follows.

1. Run PHASE for the 10,000 selected regions.
2. Examine the best haplotypes and its probability ($\max P$) for each region.
3. Calculate the precision for $\ln(1.001 - \max P)$, which is defined by

$$\mathrm{CP}_{p}/(\mathrm{CP}_{p} + \mathrm{IP}_{p}) ,$$

where p is the target $\ln(1.001 - \max P)$, and CP_p and IP_p are the number of consistent pairs and inconsistent pairs in the regions for which $\max P$ satisfy $p - 0.5 < \ln(1.001 - \max P) \leq p$.

In our evaluation, PHASE precision is more than 0.90 for $\ln(1.001 - \max P) \leq -2.5$.
